# Deficiency in the Essential Amino Acids l-Isoleucine, l-Leucine and l-Histidine and Clinical Measures as Predictors of Moderate Depression in Elderly Women: A Discriminant Analysis Study

**DOI:** 10.3390/nu13113875

**Published:** 2021-10-29

**Authors:** Silvia Solís-Ortiz, Virginia Arriaga-Avila, Aurora Trejo-Bahena, Rosalinda Guevara-Guzmán

**Affiliations:** 1 Departamento de Ciencias Médicas, División de Ciencias de la Salud, Campus León, Universidad de Guanajuato, León, Guanajuato 37320, Mexico; aurora.st@gmail.com; 2 Facultad de Medicina, Universidad Nacional Autónoma de México, Ciudad de México 04510, Mexico; varriaga@servidor.unam.mx (V.A.-A.); rguevara@servidor.unam.mx (R.G.-G.)

**Keywords:** depression, cognition, symptoms, elderly, amino acids, comorbidities

## Abstract

Increases in depression are common in some elderly women. Elderly women often show moderate depressive symptoms, while others display minimal depressive symptoms. These discrepancies have produced contradictory and inconclusive outcomes, which have not been explained entirely by deficits in neurotransmitter precursors. Deficiency in some amino acids have been implicated in major depression, but its role in non-clinical elderly women is not well known. An analysis of essential amino acids, depression and the use of discriminant analysis can help to clarify the variation in depressive symptoms exhibited by some elderly women. The aim was to investigate the relationship of essential amino acids with affective, cognitive and comorbidity measures in elderly women without major depression nor severe mood disorders or psychosis, specifically thirty-six with moderate depressive symptoms and seventy-one with minimal depressive symptoms. The plasma concentrations of nineteen amino acids, Beck Depression Inventory (BDI) scores, Geriatric Depression Scale (GDS) scores, global cognitive scores and comorbidities were submitted to stepwise discriminant analysis to identify predictor variables. Seven predictors arose as important for belong to the group based on amino acid concentrations, with the moderate depressive symptoms group characterized by higher BDI, GDS and cognitive scores; fewer comorbidities; and lower levels of l-histidine, l-isoleucine and l-leucine. These findings suggest that elderly women classified as having moderate depressive symptoms displayed a deficiency in essential amino acids involved in metabolism, protein synthesis, inflammation and neurotransmission.

## 1. Introduction

According to the World Health Organization [1], depressive symptoms include feelings of sadness, hopelessness, pessimism, and low self-esteem; a decrease in or loss of ability to feel pleasure; reduced energy and vitality; slowness of thought; loss of appetite and disturbed sleep or insomnia. These depressive symptoms are prevalent among elderly individuals [2], with a higher incidence in elderly women [3]. In some elderly women, the symptoms extend from mild to severe and need medication [4], whereas in other elderly women, symptoms are minor or absent, maybe representing the common depressive symptoms of typical elderly women [5]. Affective studies have demonstrated that for some elderly women, severe symptoms of depression may be difficult to recognize because they may exhibit different symptoms than in younger people [1]. For some elderly women with depression, sadness is not their main symptom, they may have other, less obvious symptoms of depression, probably related to other comorbidities and cognitive impairment [6]. Depression in elderly individuals is multifactorial, which makes its assessment difficult [7]; however, deficiencies in some amino acids involved in brain metabolism can be a relevant factor for alterations in mood [8]. Some studies have suggested an association between plasma amino acids and psychosis in mixed populations [9]. Individuals with the first episode of psychosis had reduced γ-aminobutyric acid (GABA) plasma levels [10] and decreased levels of proline, alpha-aminoadipic acid, kynurenine, valine, tyrosine, citrulline, tryptophan, and histidine compared to controls [11]. Another study found low levels of tryptophan associated with major depression [12]. Some studies have also reported low levels of tryptophan, methionine, phenylalanine, and tyrosine [13,14], as well as GABA, dopamine, tyramine and kynurenine in patients with major depression compared to controls [15]. Branched-chain amino acids have been implicated in major depression, although they have not been extensively analyzed. Low levels of leucine were reported in patients diagnosed with bipolar disorder [16], whereas leucine, valine and isoleucine levels were decreased in middle-aged patients of both genders with major depression [17]. However, most results related to biomarkers are inconsistent due to the heterogeneity of depression and the diverse methodology used, such as the inclusion of individuals of different ages, sexes and diagnoses [18].

Depressive symptoms are not sufficiently characterized in elderly women over 65 years of age because there are elderly women who show moderate depressive symptoms, while others display minimal depressive symptoms. According to the American Psychiatric Association, minor depressive symptoms are characterized by symptoms that often pass unnoticed because the presence of depressed mood is not detected, nor the inability to enjoy things. In contrast, moderate depressive symptoms are characterized by the manifestation of symptoms that produce some difficulty in continuing with activities of daily living, whose intensity may cause discomfort but is manageable. These discrepancies have produced ambiguous outcomes, which have not been resolved completely by factors related to depression. Most studies have used correlation analysis to identify relationships between depressive scores and declining amino acid levels in individuals of different ages who were diagnosed with major depression. In some investigations, this approximation has provided appropriate understanding, but other investigations have informed conflicting outcomes. The interpretation of correlation analysis is suitable but limited when one demands to detect the features that differentiate two groups and to establish a function capable of discern between the members of the two groups with the large feasible accuracy. Discriminant analysis allows the prediction of group membership from a set of predictors (independent variables) separating these variables from others that are orthogonally independent [19]; hence, discriminant analysis is an appropriate statistical method to detect the variables that allow differentiation between groups and to establish how many of these variables are required to reach the optimal feasible categorization [20]. An examination of the discriminant function coefficients of amino acid profiles and affective, global cognition and comorbidity measures between moderately and minimally depressed women, undiagnosed with major depressive disorder, by discriminant analysis may help us in enhancing our comprehension of the impact of amino acid deficiencies on depressive symptoms.

The aim of the present research was to examine the amino acid profiles and the mood, global cognitive and comorbidity measures in elderly women with moderate and minimal levels of depressive symptoms, to identify, through discriminant analysis, predictor variables that best classify subjects. This classification could help to elucidate the variation in depressive symptoms shown by some elderly females related with optimal or deficiencies in amino acid concentrations. We expected that the moderate depressive symptoms group would exhibit profile variables that could reflect alterations in mood based on depressive symptom scores, global cognitive scores, the number of comorbidities and plasma amino acid profiles.

## 2. Materials and Methods

### 2.1. Participants

Ninety-seven elderly women between 65 and 79 years of age participated in this cross-sectional study. The elderly women were recruited from a senior center. This center provides health care to elderly women through recreational and occupational activities. The study only included a sample of elderly women without signs of pre-existing severe mental disorders, according to the Diagnostic and Statistical Manual of Mental Disorders, 5th Edition (DSM-5) [21]. Women with severe major depression, bipolar disorder, dysthymia, dysphoria or psychosis such as schizophrenia were excluded from the study. Therefore, this is a non-clinical sample that describes the depressive symptoms of a group of elderly women attending a senior center. To estimate the statistical power of the sample size of 97 women, the mean and standard deviations were obtained. The BDI [22] depressive scores for each group were used to separate the patients into two groups. A power of 0.80 was regarded to identify a 10% contrast in BDI scores, with a significance level of α = 0.05. The power of this sample size was appropriate to continue with the examination of the variables of the current research, with a large effect size (Cohen’s d = 0.83). Since females show more susceptibility to depression [23], only females were selected for this investigation. The elderly women were divided into a minimal depressive symptoms group (*n* = 61) and a moderate depressive symptoms group (*n* = 36) according to their scores of depressive symptoms on the BDI questionnaire. So, the two groups were contrasted. The stratification criterion was based on these punctuations: 61 elderly women who scored between 0 and 9 were classified in the minimal depressive symptoms group, whereas 36 elderly women who scored between 10 and 29 were classified in the moderate depressive symptoms group. Thus, a high score was indicative of moderate depressive symptoms [5]. The elderly women were examined in a unique session (between 0900 h and 1000 h) by one trained investigator, who asked them about their additional illnesses, medications and hormone replacement therapy. The elderly women included in the study were not treated with antidepressant. This research was approved by the Research and Ethics Committee of the Faculty of Medicine of the National Autonomous University of Mexico (No. 007-2018). All of the elderly females conceded written informed consent before to participate in the investigation.

### 2.2. Depression Questionnaires

The BDI questionnaire [22], in a standardized version for the Mexican population [24], was used to evaluate self-reports of current depressive symptoms in 97 elderly women. The BDI questionnaire consists of 21 items that measure current depressive symptoms. Each item contains a group of four statements, from which the subject chooses one according to how she felt in the last week. These statements reflect the severity of the discomfort produced by depressive symptoms and are marked from 0 (minimal) to 4 (severe). The total score of scale is obtained by adding the scores for the 21 items, with 0 as the lowest score and 64 as the maximum score. Individual questions of the BDI assess mood, pessimism, sense of failure, self-dissatisfaction, guilt, punishment, self-dislike, self-accusation, suicidal ideas, crying, irritability, social withdrawal, body image, work difficulties, insomnia, fatigue, appetite, weight loss, bodily preoccupation, and loss of libido. Items 1 to 13 assess symptoms that are psychological in nature, while items 14 to 21 assess more physical symptoms. In the present study, we used the cut-off points to categorize the severity of depression according to the version of the BDI questionnaire adapted for the Mexican population [24]. A score of 0 to 9 reflected the absence or minimal presence of depressive symptoms; 10 to 16 indicated a medium depression; 17 to 29 reflected moderate depression and scores of 30 to 63 indicated severe depression. Since only one elderly woman scored in medium depression, she was aggregated with the elderly women who scored in the 17 to 29 range, so scores from 10 to 29 were considered moderate depressive symptoms. Furthermore, one of the participants who scored in the range of 30 to 35 was excluded from the study. Depressive scores were compared between groups with minimal and moderate symptoms.

The GDS questionnaire [25], in a standardized version for the Spanish-speaking population [26], was also used to identify symptoms of depression in elderly women. The GDS questionnaire evaluates sadness, lack of energy, positive mood, social withdrawal, agitation, feelings of worthlessness, feelings of despair and perception of cognitive changes. Each item on the GDS is scored with one point for a depressive response. Items are summed to determine the total score with a maximum of 30 points. Scores ranging from 0 to 9 indicate normal mood, scores of 10 to 19 indicate mild depressive symptoms, and scores of 20 to 30 indicate severe depressive symptoms.

### 2.3. Dementia and Global Cognition

Initial dementia and general cognition were assessed employing the Mini-Mental State Examination (MMSE) [27] in a standardized version for the Spanish-speaking population [28] to establish cognitive status in different areas that can be related to different cognitive symptoms. These cognitive symptoms include temporal and spatial orientation, immediate memory and retention, concentration and working memory, language and graphic constructive praxis. Possible scores on this examination range from 0 to 30, and subjects with dementia usually score under 24.

### 2.4. Quantification of Amino Acids

Before the depressive symptom evaluation period, a 10 mL blood sample was obtained from an antecubital vein from the women between 0800 h and 0900 h after a nocturne fast. Blood samples were centrifuged at 25 °C to get plasma. The plasma was accumulated, distributed into 2 mL tubes, and stored at −80 °C until examination. The quantification of the amino acids in the plasma was performed using an Agilent 1100 HPLC system (Agilent Technologies, Santa Clara, CA, USA) based on the procedure reported by Henderson et al. [29]. The following 19 amino acids were quantified with this method: l-aspartate, l-glutamate, l-asparagine, l-serine, l-glutamine, l-histidine, l-glycine, l-threonine, l-citrulline, l-arginine, l-alanine + taurine, GABA, l-tyrosine, l-valine, l-methionine, l-tryptophan, l-phenylalanine, l-isoleucine and l-leucine.

### 2.5. Discriminant Analysis

The current investigation executed stepwise discriminant analysis [19] to create a model to prognosticate which groups the subjects belong to. Also, to determine predictor variables of moderate and minimal depressive symptoms in elderly women based on their BDI depressive scores, GDS depressive scores, MMSE scores, comorbidities and amino acid profiles. We employed discriminant analysis because we were focused in detecting prognostic variables of group membership [30]; therefore, the discriminant analysis was executed after the females were divided into moderate and minimal depressive symptom groups.

### 2.6. Statistical Procedure

Statistical procedure and the power of the sample were determined with the Statistical Package for the Social Sciences (SPSS) version 23, IBM, New York, NY, USA. We used a two-tailed α value of 0.05 and a β value of 0.80 to calculate a standardized effect size of 10% based on the BDI scores between groups. Prior to statistical procedures were employed, the data were inspected for distribution normality applying the Kolmogorov-Smirnov test. Partial Wilks’ lambda was used to compare the means of the characteristics of the elderly women and their amino acid profiles between the two groups. A stepwise multiple discriminant analysis [19] was executed to detect latent variables as predictors of moderate and minimal depressive symptoms in elderly women based on their BDI depressive scores, GDS depressive scores, MMSE scores, comorbidities, and plasma concentrations of amino acids. Contrasts were considered as significant when *p* was <0.05.

## 3. Results

### 3.1. Characteristics of the Participants and Depressive Symptoms

Sample characteristics included age, years of schooling, age at menarche, age at menopause, weight, height, body mass index, systolic arterial pressure, diastolic arterial pressure, comorbidities, BDI depression scores, GDS depression scores and MMSE scores, which are presented in Table 1. The results show the test of equality of group means with the coefficients of the partial Wilks’ lambda derived from the discriminant analysis. The BDI and GDS depression scores were significantly elevated in the moderate-depressive group, which indicated that this group scored between 10 and 29 on the BDI and between 9 and 14 on the GDS, respectively. None of the elderly women had severe depressive symptoms. There were no noteworthy contrasts among the minimal and moderate depressive symptoms groups in the rest of their features or in their dementia indexes or comorbidities. The most prevalent comorbidities reported by the elderly women included hypertension (63%), osteoporosis (69%), arthritis (52%), gynecological disease (78%), surgery (94%) and kidney disease (67%). Other diseases (62%) included glaucoma, migraine, hepatitis, hypothyroidism, diabetes, allergies and sinusitis.

### 3.2. Amino Acid Profiles

The plasma amino acid profiles of the minimal and moderate depressive symptom groups are presented in Table 2. The results show the test of equality of group means with the coefficients of the partial Wilks’ lambda derived from the discriminant analysis. Plasma concentrations of l-methionine were elevated in the moderate depressive symptom groups, while l-isoleucine levels were elevated in the minimal depressive symptom groups. l-Aspartate, l-glutamate, l-asparagine, l-serine, l-glutamine, l-histidine, l-glycine, l-threonine, l-citrulline, l-arginine, l-alanine + taurine, GABA, l-tyrosine, l-valine, l-methionine, l-tryptophan, l-phenylalanine and l-leucine did not display notable contrasts between the moderate and minimal depressive symptom groups. Discriminant analysis revealed additional elements, which are exposed below.

### 3.3. Discriminant Analysis

The discriminant analysis results are displayed in Table 3. Discriminant analysis detected seven latent variables as predictors of moderate and minimal depressive symptoms: BDI scores, GDS scores, MMSE scores, comorbidities, l-histidine, l-isoleucine, l-leucine and one discriminant function, which described 100% of the variance, canonical = 0.864. The canonical correlation value was squared to estimate the effect size of the discriminant function, R^2^ = 0.74, which showed a large effect size. This discriminant function significantly differentiated the depressive groups, Wilks’s lambda = 0.253; *χ^2^* = 127.20, df = 7, *p* < 0.001. The small lambda indicates that the group means diverge. The eigenvalue was great at 2.956, showing that the function differentiates well among the groups, and the canonical correlation was near to 1 (0.86). Standardized canonical discriminant function coefficients showed that BDI scores, GDS scores, MMSE scores, comorbidities and l-isoleucine had a positive relationship, whereas l-histidine and l-leucine had a negative relationship with this function. High punctuations demonstrate that a dependent variable is crucial for a variate, and variables with positive and negative coefficients contribute to the variate in opposite ways [30]. Structure matrix coefficients showed that BDI scores (0.82) and GDS scores (0.36) had high canonical correlations and contributed the most to group separation, whereas l-isoleucine (0.14), MMSE scores (0.06), l-leucine (0.04) and comorbidities (0.02) contributed less and l-histidine (−0.06) contributed in the opposite manner. High canonical variate correlations indicate that these variables contribute the most to group separations.

The estimate of the functions at group centroids distinguished the centroids of the moderate-depressive symptoms group, which were situated in the positive zone (2.185), whereas the centroid of the minimal-depressive symptoms group was situated in the negative zone (−1.325). These outcomes showed that the groups with values opposite in sign were discriminated by the functions. Of the 61 elderly women who were in the minimal-depressive symptoms group, 61 (100%) were correctly classified as members of that group, and the 36 who were in the moderate-depressive symptoms group, 36 were also correctly classified supported on the selected variables.

The outcomes of classification function coefficients in moderate- and minimal-depressive symptom groups supported on Fisher´s linear discriminant functions are displayed in Table 4. These coefficients mention us between which of the groups the specific functions discriminate and permit us to establish to which group each subject most probable belongs. These coefficients revealed that the moderate-depressive symptoms group displayed high coefficients in the predictor variables, which suggests that this group had higher BDI, GDS, and MMSE scores; fewer comorbidities; and reduced levels of l-histidine, l-isoleucine, and l-leucine contrasted to the minimal-depressive symptoms group.

Our results showed, through the structure matrix, that the coefficients with high and low canonical correlations were used to establish group membership. They seem to indicate some associations. However, a causality cannot be established with this analysis, so future studies should consider the inclusion of other analysis, methods and variables.

## 4. Discussion

The current study identified seven factors as significant predictors of group membership based on amino acid concentrations, depression scores and global cognitive scores, with the moderate depressive symptoms group characterized by higher BDI, GDS scores, and cognitive scores; fewer comorbidities; and lower levels of l-histidine, l-isoleucine and l-leucine. These results indicate that elderly women classified in the moderate depressive symptoms group exhibited deficiencies in essential amino acids in addition to self-report of depressive symptoms. Our outcomes also revealed that patients with minimal depressive symptoms had optimal concentrations of essential amino acids. This could explain, at least in part, the moderate depressive symptoms exhibited by some elderly females. Moreover, discriminant analysis successfully classified group membership; hence, the differences between the groups could be established. The present study did not perform additional analyzes to find definitive causal relationships between essential amino acids and depression. However, the discriminant analysis provides a multivariate analysis that include χ^2^, canonical correlations, and through the structure matrix, the coefficients with high and low canonical correlations are obtained and used to establish group membership associations [19], based on the essential amino acids and depression.

The present study showed that BDI and GDS depressive scores were two variables that emerged as predictors of depression in elderly women classified into the moderate depressive symptoms group. This group exhibited elevated depressive symptoms on the BDI [22] and GDS [25] questionnaires, suggesting moderate depressive symptoms according to their normative data. The elderly women analyzed here had high scores on the BDI questionnaire, mainly for items that evaluated negative thoughts and attitudes, which could contribute to the maintenance of their depressed mood [31]. GDS evaluates depressive symptoms in the elderly people. However, some factors share similarities with BDI, such as sadness, lack of energy, social withdrawal, feelings of worthlessness or despair. In our study, it was notable to observe that BDI and GDS depression scores were higher in the moderate depressive symptoms group. This suggests that the intensity of depressive symptoms was detected in a similar way by both questionnaires. In a sample with similar characteristics, moderate depressive symptoms were also reported in middle-aged women without major depression [5], which is consistent with our results.

In the current research, comorbidities were another variable that arose as a predictor. The comorbidities were higher in the minimal depressive symptoms group and lower in the moderate depressive symptoms group. The most prevalent comorbidities reported by the elderly women included hypertension, osteoporosis, arthritis, gynecological disease, surgery and kidney disease. Other diseases included glaucoma, migraine, hepatitis, hypothyroidism, diabetes, allergies and sinusitis. These comorbidities have been related to depressive symptoms [6,32], which partially coincides with our outcomes. These multiple comorbidities found in elderly women analyzed here makes them delicate individuals. A study conducted in elderly hospitalized individuals demonstrated that malignancy, diabetes, coronary artery disease, chronic kidney disease and chronic obstructive pulmonary disease were more frequent in men, but hypertension, osteoarthritis, anemia and depression were more frequent in women [33]. These findings coincide and support our results, even though we had not considered men in our study, seem to highlight the gender differences that impact the development of diseases in vulnerable elderly. Another study identified different frailty phenotypes, differently associated with adverse events, as multimorbidity and cognitive impairment in old patients [34], which is consistent our outcomes. Some studies have reported certain protective factors associated with depressive symptoms in older adults. Social support to manage health problems and the role engagement in social activities, volunteer work or religion have a significant influence on the manifestation of depression [35]. One study found that the hobbies and indoor activities was associated with lower odds of elevated symptoms for men and women, while the volunteer and community activities was associated with lower odds of depressive symptoms for women [36]. In our study, elderly women attended a senior center that provides recreational and occupational activities, which could influence depression and comorbidities. It has been suggested that resilience in some elderly individuals can support adaptation to comorbidities for successful aging [37], which could explain, at least in part, our findings in the moderate depressive symptoms group.

In the present study, MMSE scores were other variable that emerged as a predictor of moderate depressive symptoms. It has been reported that depressive symptoms predict cognitive decline in elderly individuals [38,39]. Specifically, a decrease in scores for global cognitive function (MMSE) and cognitive flexibility was associated with depressive symptoms in nondemented elderly women [40]. In addition, elderly individuals with severe depression had twice risk for cognitive dysfunction [41]. These results partially support our findings found in the moderate depressive symptoms group. Furthermore, in our research, the elderly women were recruited from a senior center, which provides health care and recreational activities. In this non-clinical sample, no signs of psychosis or other states such as bipolar disorders were detected. Therefore, the results in the MMSE could not be influenced by these disorders. Moreover, it was remarkable to observe that the scores obtained in the MMSE did not show significant differences between the two study groups. However, the discriminant analysis established suitable group membership for MMSE scores.

Notably, in our investigation, l-isoleucine and l-leucine were two essential amino acids that emerged as predictors of moderate depressive symptoms. l-Isoleucine is an essential amino acid that the body cannot manufacture, so it must be obtained through the diet. The benefits of l-isoleucine may include regulating blood glucose, participating in hemoglobin synthesis, and reducing postworkout fatigue and central fatigue [42,43]. l-Leucine is an activator of the mammalian target of rapamycin that regulates protein synthesis, tissue regeneration and metabolism [44]. In the brain, l-leucine serves as a metabolic precursor of fuel molecules that are forwarded by astrocytes to adjacent neural cells for brain energy metabolism [45]. l-Leucine also participates in the regulation of the neurotransmitter glutamate [46]. The moderate depressive symptoms group of elderly women analyzed here had less l-isoleucine and less l-leucine, suggesting deficits in protein synthesis and brain energy metabolism. These deficits also suggest alterations in neurotransmitter glutamate and glucose metabolism, which are crucial to brain function and neural cell vitality [47]. These deficits could explain, at least in part, our outcomes found in elderly women with moderate depressive symptoms. One study revealed that patients diagnosed with bipolar disorder and major depression showed low levels of leucine and isoleucine, and these amino acids were negatively correlated with depressive scores [16,17], which partially supports our findings.

In our investigation, l-histidine was another amino acid that emerged as a predictor of moderate depressive symptoms. l-Histidine is an essential amino acid that has a role in protein components and is a precursor of histamine involved in inflammation. Brain histamine is synthesized from l-histidine in the presence of histidine decarboxylase to play a role as a neurotransmitter in the brain to balance mood, the sleep cycle, learning, memory, alertness, regulation of appetite and the perception of pain [48]. Since histamine is required for many brain processes, low levels of histamine can manifest as depression, anxiety and poor motivation [48], probably related to a reduction in histamine receptors [49]. The moderate depressive symptoms group of elderly women analyzed here had less l-histidine, suggesting deficits in brain histamine, which could induce depressive symptoms. Our outcome is partially supported by a study that showed that histidine intake improved depression, attentiveness, concentration and mental task performance [50].

The findings of the present study have implications for clinical practice in the development of strategies for the prevention of depressive symptoms associated with protein deficiency in vulnerable older adults. Our results will also serve to apply treatments with balanced diets to decrease the manifestation of depressive symptoms in elderly individuals. However, the present study has limitations: First, we did not include elderly women diagnosed with major depression that could help support the effects of essential amino acids in severe depression. Second, we did not evaluate the food intake of elderly women that could help clarify the relationship with protein intake and depressive symptoms. Third, we did not include elderly men without major depression to establish comparisons with gender. Fourth, we did not include elderly women without depression to establish comparisons with the group studied here with the moderate depressive symptoms, which should be included in future studies. The strength of this study is that we used a discriminant analysis to establish deficiencies in essential amino acids related to moderate depressive symptoms, which could reflect alterations in nutrition. Even though the present study did not measure food intake, protein deficiency has been associated with depression in older adults [51] and middle-aged individuals [52]. These results partially support our findings found in elderly women. Since essential amino acids, l-isoleucine and l-leucine, serve as signaling molecules regulating metabolism of glucose, lipid, and protein synthesis, and immunity, a dietary optimizing of these essential amino acids will have health benefits [53].

## 5. Conclusions

In summary, our results emphasize that the moderate depressive symptoms group was characterized by higher BDI, GDS, and global cognitive scores; fewer comorbidities; and less concentration l-histidine, l-isoleucine and l-leucine. These results suggest that this group showed a deficiency in essential amino acids involved in metabolism, protein synthesis, inflammation and brain neurotransmission, which could influence depressive symptomatology. This insufficiency could be related to nutritional deficiencies, particularly protein intake.

## Figures and Tables

**Table 1:** Features of the elderly women with minimal and moderate depressive symptoms.

Features	Minimal Mean ± SD	Moderate Mean ± SD	Partial Wilks’ Lambda	*F* Value	*p* Value
Age (years)	66 ± 6.0	67 ± 5.7	0.993	0.67	0.41
Years of education	8 ± 5.6	6 ± 4.0	0.976	2.35	0.12
Age at Menarche	12 ± 1.0	13 ± 1.0	0.992	0.958	0.35
Age at Menopause	48 ± 3.9	47 ± 4.0	0.998	−1.022	0.25
Weight (kg)	72 ± 13.0	68 ± 7.0	0.981	−0.426	0.10
Size (m)	1.5 ± 0.10	1.5 ± 0.10	0.978	−0.805	0.92
BMI (kg/m^2^)	30 ± 5.0	28 ± 3.0	0.973	0.112	0.91
TAS (mmHg)	113 ± 11.0	127 ± 9.0	0.992	0.335	0.40
TAD (mmHg)	70 ± 6.0	75 ± 5.0	0.966	0.633	0.08
Comorbidities	2 ± 0.9	2 ± 1.0	0.998	0.22	0.64
GDS depression scores	3 ± 3.6	9 ± 5.7	0.715	38.20	0.0001
BDI depression scores	4.5 ± 2.6	18 ± 6.6	0.331	194.00	0.0001
MMSE scores	24.5 ± 4.3	26 ± 3.1	0.987	1.24	0.27

MMSE = Mini Mental State Examination; BMI = Body Mass Index; TAS = Systolic arterial tension; TAD = Diastolic arterial tension.

**Table 2:** Amino-acid profiles (µM) of the elderly women with minimal and moderate depressive symptoms.

Amino-Acids	Minimal Mean ± SD	Moderate Mean ± SD	Partial Wilks’ Lambda	*F* Value	*p* Value
l-Aspartate	3.2 ± 4.2	4.2 ± 3.9	0.986	1.367	0.24
l-Glutamate	166.6 ± 153.2	173.6 ± 90.5	0.999	0.066	0.79
l-Asparagine	27.2 ± 7.0	26.6 ± 7.0	0.998	0.157	0.69
l-Serine	105.3 ± 24.8	110.2 ± 18.4	0.988	1.164	0.28
l-Glutamine	383.2 ± 139.2	361.8 ± 102.3	0.993	0.656	0.40
l-Histidine	62.0 ± 10.8	59.8 ± 8.3	0.988	1.158	0.28
l-Glycine	272.6 ± 74.2	251.9 ± 72.0	0.981	1.825	0.18
l-Theonine	104.9 ± 24.4	107.5 ± 28.8	0.998	0.237	0.62
l-Cirtrulline	23.8 ± 6.5	25.2 ± 7.1	0.990	1.007	0.31
l-Arginine	48.0 ± 21.3	50.7 ± 20.0	0.996	0.386	0.53
l-Alanine + Taurine	195.4 ± 45.3	192.8 ± 39.8	0.999	0.086	0.77
GABA	2.2 ± 2.0	2.6 ± 2.8	0.991	0.916	0.34
l-Tyrosine	58.6 ± 12.7	63.1 ± 16.9	0.979	2.009	0.16
l-Valine	182.8 ± 27.8	192.8 ± 32.3	0.973	2.635	0.10
l-Methionine	25.3 ± 8.0	28.7 ± 9.7	0.966	3.429	0.05
l-Tryptophan	32.7 ± 7.1	34.5 ± 9.7	0.984	1.552	0.21
l-Phenylalanine	51.5 ± 8.5	51.5 ± 8.1	1.000	0.002	0.96
l-Isoleucine	55.9 ± 14.5	49.3 ± 11.7	0.939	6.232	0.01
l-Leucine	123.0 ± 22.0	119.5 ± 20.2	0.993	0.651	0.42

GABA = γ-aminobutyric acid

**Table 3:** Outcomes of standardized canonical discriminant function coefficients and predictor variables ordered by absolute size of correlation within function.

Predictor Variables	Standardized Coefficients	Structure Matrix	*F* Ratio	*p* Value
BDI depression scores	0.961	0.827	194.00	0.0001
MMSE scores	0.386	0.066	102.35	0.0001
Comorbidities	0.396	0.028	74.60	0.0001
l-Histidine	−0.196	−0.064	58.87	0.0001
GDS depression scores	0.333	0.367	49.34	0.0001
l-Isoleucine	0.508	0.148	42.39	0.0001
l-Leucine	−0.396	0.048	38.00	0.0001
Canonical correlation	0.864			
Effect size R^2^	0.74			
Eigenvalue	2.956			
Wilks’ lambda	0.253			
*x* ^2^	127.20			0.0001

GDS = Geriatric Depression Scale; BDI = Beck Depression Inventory; MMSE = Mini Mental State Examination.

**Table 4:** Outcomes of classification function coefficients in moderate and minimal depressive symptom groups.

Predictors	Moderate Symptoms	Minimal Symptoms
BDI depression scores GDS depression scores MMSE scores	0.579	−0.157
	1.201	0.943
	2.243	1.903
Comorbidities l-histidine	0.276	1.658
	0.365	0.434
l-isoleucine l-leucine	0.019	−0.120
	0.220	0.286
Fisher´s linear discrimination functions		
